# Effect of Trehalose and Ceftriaxone on the Stability of Aggregating-Prone Tau Peptide Containing PHF6* Sequence: An SRCD Study

**DOI:** 10.3390/ijms23062932

**Published:** 2022-03-08

**Authors:** Claudia Honisch, Federica Torni, Rohanah Hussain, Paolo Ruzza, Giuliano Siligardi

**Affiliations:** 1 Institute of Biomolecular Chemistry of CNR, Padua Unit, Via Marzolo 1, 35131 Padova, Italy; c.honisch@icb.cnr.it (C.H.); tornifederica@gmail.com (F.T.); 2 Department of Chemical Sciences, University of Padua, Via Marzolo 1, 35131 Padova, Italy; 3 Diamond Light Source Ltd., Harwell Science and Innovation Campus, Didcot, Oxfordshire OX11 0DE, UK; rohanah.hussain@diamond.ac.uk

**Keywords:** tau protein, intrinsically disordered proteins, tauopathies, protein aggregation, trehalose, ceftriaxone, conformational stability, synchrotron radiation circular dichroism, transmission electron microscopy

## Abstract

The tau protein, a soluble protein associated with microtubules, which is involved in the assembly and stabilization of cytoskeletal elements, was found to form neurofibrillary tangles in different neurodegenerative diseases. Insoluble tau aggregates were observed to be organized in paired helical filaments (PHFs) and straight filaments (SFs). Recently, two small sequences (306–311 and 275–280) in the microtubule-binding region (MTBR), named PHF6 and PHF6*, respectively, were found to be essential for tau aggregation. Since a possible therapeutic approach consists of impairing amyloid formation either by stabilizing the native proteins or reducing the level of amyloid precursors, here we use synchrotron radiation circular dichroism (SRCD) at Diamond B23 beamline to evaluate the inhibitory effects of two small molecules, trehalose and ceftriaxone, against the aggregation of a small peptide containing the PHF6* sequence. Our results indicate that both these molecules, ceftriaxone and trehalose, increased the stability of the peptide toward aggregation, in particular that induced by heparin. With trehalose being present in many fruits, vegetables, algae and processed foods, these results support the need to investigate whether a diet richer in trehalose might exert a protective effect toward pathologies linked to protein misfolding.

## 1. Introduction

Intrinsically disordered proteins (IDPs) are a widespread class of proteins with the ability to quickly change their conformations upon participating in biological processes [1]. IDP structures are highly controlled in the cell, and aberrant regulation is often associated with protein aggregation and human diseases [2].

The tau protein, a soluble protein associated with microtubules whose function is to assist the assembly and stabilization of microtubules and other cytoskeletal elements, represents an archetypical IDP found in the nervous system [3].

Neurofibrillary tangles of tau protein occur in neurons and glial cells of patients affected by different neurodegenerative diseases known as tauopathies, including, but not limited to, Alzheimer’s disease, progressive supranuclear palsy, corticobasal syndrome, some frontotemporal dementias and chronic traumatic encephalopathy [4]. A strict correlation has been found between the extent and anatomical localization of tau aggregates and the progression of Alzheimer’s disease [5]. At the ultrastructural level, tau aggregates are assembled in insoluble paired helical filaments (PHFs) and straight filaments (SFs) [6]. These aggregates typically show a cross-β pattern that is common to many aggregated proteins [7,8] showing an ordered β-sheet amyloid core flanked by an unstructured coat.

**Figure 1:**
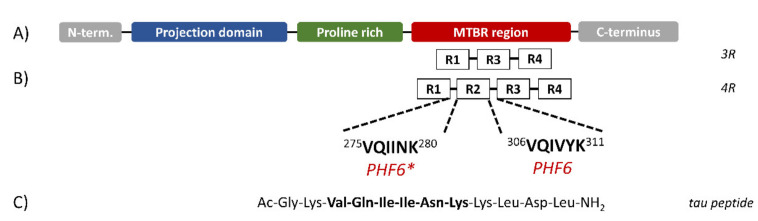
Schematic structure of Tau isoforms. (**A**) Tau protein can be divided in three domains: the N-terminal projection domain containing two alternatively spliced exons; a proline-rich region; the microtubule-binding region (MTBR). (**B**) The MTBR region may contain three (*3R*) or four (*4R*) pseudo-repeat units (R1 to R4) separated by short inter-repeat regions where the PHF6 and PHF6* segments are located. (**C**) Sequence of the synthesized peptide. (Figure redrawn from Ref. [15]).

Six isoforms of tau protein have been isolated in adult human brain containing either three or four pseudo-repeat units (3R or 4R, respectively) separated by short inter-repeat regions (Figure 1) [9], both forming the microtubule-binding region (MTBR). Recently, two small sequences in the MTBR region, corresponding to the sequences 306–311 and 275–280, named PHF6 and PHF6*, respectively, were found to be essential for tau aggregation [10] (Figure 1B). The importance of the last sequence to tau aggregation has been confirmed, as its deletion slowed down the formation of aggregates [11], whilst its presence in 4R proteins, but not in 3R proteins, increased the propensity to aggregate in vitro [12]. Moreover, several tau mutations found in frontotemporal dementia are located within the PHF6* sequence [13].

Several research groups have found that short peptides derived from tau protein containing the PHF6* region [14,15] form oligomeric structures rich in β-structure rather than full-fledged pairs of helical filaments. These observations indicate that the PHF6* sequence appears to play a key role in nucleating the aggregation, while other tau segments generate the PHF morphology.

The aggregation of tau protein and its fragments in vitro can be induced by several agents such as arachidonic acid, RNA, and heparin. Although the question of whether heparin-induced tau aggregation is pathologically relevant is far from been resolved, it is important to note that heparin is closely related to heparansulfate that has been found in neurofibrillary tangles in Alzheimer’s disease lesions [16]. Moreover, when associated with proteoglycans, heparansulfate mediates the cellular internalization and propagation of tau aggregates via a prion-like mechanism [17].

One approach in the therapy of Alzheimer’s disease and other neurodegenerative pathologies characterized by the presence of protein aggregates is to block or impair amyloid formation either by stabilizing the native proteins, reducing the level of amyloid precursors or increasing the clearance of insoluble aggregates. In this context, several natural and synthetic compounds have been investigated as possible candidates [18,19,20]. Recently, we demonstrated that trehalose and ceftriaxone (Figure S1) may act as inhibitors of protein aggregation [21,22,23,24]. Ceftriaxone was able to successfully eliminate the cellular toxic effects of misfolded glial fibrillary acid protein (GFAP) in a cellular model of Alexander’s disease [25] as well as to protect dopaminergic neurons ameliorating motor deficits in a rat model of Parkinson’s disease [26]. On the other hand, trehalose, a non-reducing glucose disaccharide acting as an osmolyte that protects cells, prevented protein denaturation [27,28] interfering with protein misfolding and subsequent aggregation [29].

Circular dichroism spectroscopy is the ideal tool to evaluate the capability of small molecules to interfere with protein aggregation [30,31], directly monitoring the protein/peptide structural changes as they undergo aggregation.

Here, we used synchrotron radiation circular dichroism (SRCD) at Diamond B23 beamline to evaluate the inhibitory effects of trehalose and ceftriaxone against the aggregation of a small peptide containing the PHF6* sequence (Figure 1C). The results indicate that in the presence of heparin, the peptide adopted a β-sheet structure forming amyloid fibrils and that this conformational transition was hampered by the addition of trehalose, whilst ceftriaxone strongly modified the fibrils’ morphologies, affecting the in vitro aggregation process.

## 2. Results and Discussion

The peptide corresponding to the wild-type sequence 273–284 (Ac-GKVQIINKKLDL-NH_2_) of the tau protein and including the PHF6* (VQIINK) region (Figure 1C) was synthesized using solid-phase peptide synthesis. Earlier work on this peptide showed that it aggregates in vitro in a qualitatively similar manner to the full-length tau protein and is consistent with the aggregating properties of longer IDPs [15,16].

### 2.1. Circular Dichroism Spectroscopy

**Figure 2:**
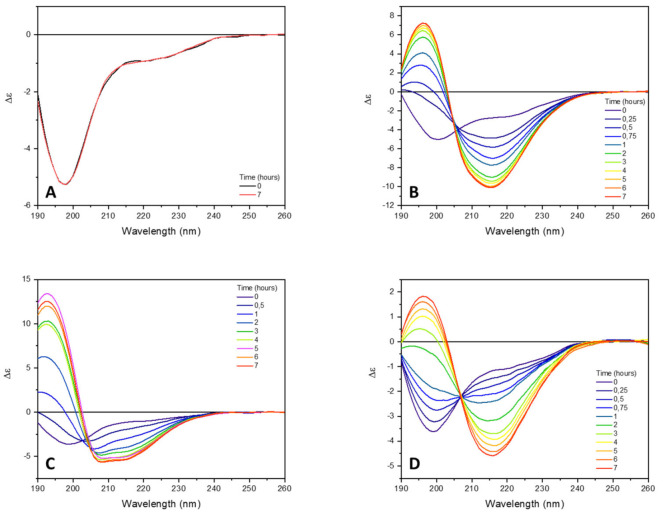
Far-UV SRCD spectra of tau peptide (70.9 µM) in 10 mM TRIS-HCl buffer, pH 7.4. (**A**) Alone; (**B**) in presence of 0.25 equivalents of low-molecular-weight heparin; (**C**) in presence of 0.25 equivalents heparin and 6 equivalents of trehalose; (**D**) in presence of 0.25 equivalents of heparin and 6 equivalents of ceftriaxone. CD spectra were recorded at different times of incubation at 25 °C (indicated).

In buffer solution, at neutral pH value, the CD spectrum of tau peptide shows the typical features of unordered conformation characterized by the presence of a negative band at about 197 nm (Figure 2). The incubation of peptide at 25 °C up to 7 h did not induce any CD change reflecting the lack of aggregates.

To induce and accelerate the tau peptide conformational transition from an unordered to β-sheet structure characteristic of peptide aggregation, low-molecular-weight heparin (0.25 equivalents) was added to the peptide solution. As shown in Figure 2B, the addition of heparin was accompanied by a progressive change in the CD spectrum of the tau peptide, coinciding with a conformational transition of the peptide from an unordered to a β-sheet conformation. Indeed, after 7 h of incubation at 25 °C, the CD spectrum of tau peptide revealed the presence of a negative band at about 216 nm and a positive CD band at about 197 nm, characteristic of a β-sheet conformation.

**Figure 3:**
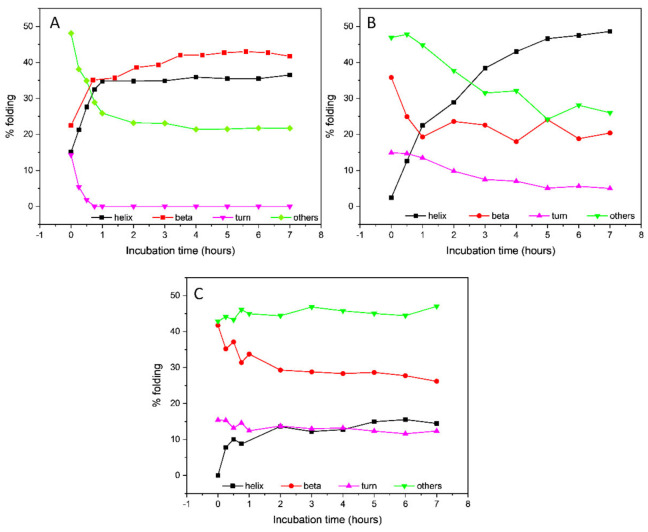
Secondary structure estimation of Tau peptide in presence of either (**A**) 0.25 eq. low-molecular-weight heparin, (**B**) 0.25 eq. heparin and 6 eq. trehalose or (**C**) 0.25 eq. heparin and 6 eq. ceftriaxone throughout 7 h incubation time.

Little or no change in the CD spectrum was observed after 7 h (data not shown), indicating that the conformational transition was completed in that time frame. The secondary structure estimation (SSE) from the CD data using the BeStSel algorithm [32,33] revealed an increase in the β-sheet content up to 41.7% (Table 1 and Figure 3), mostly attributable to the formation of a parallel β-sheet structure, accompanied by a decrease in the content of antiparallel β-sheet and unordered secondary structures. An isodichroic point at about 204 nm was consistent with a single-phase transition from the initially unordered structure of tau peptide to the final β-sheet conformation after the addition of heparin.

To better understand the conformational transition of the tau peptide in the presence of heparin, the set of acquired CD spectra was analyzed using singular value decomposition (SVD) analysis to extract the key spectral components and their kinetics. SVD decomposed the data set into three distinct sections: a basis set that represented the independent spectral features; kinetic traces for each of these features, describing their contribute to each time; and a weight value (W) that ranked the importance of each basis spectrum, providing an unbiased rationale for the reduction in the basis set dimension.

Figure S2 in the Supplementary Materials depicts six basis spectra generated by the SVD analysis implement in the CDApps software [34], their associated kinetics, and weight values. As the spectral features are merely a mathematical representation of the data, they can have both positive and negative amplitudes. The contribution of each basis spectrum fell rapidly, and only the first two spectra were utilized for analysis, being consistent with a single-phase transition. Indeed, the sum of the squares of these two singular weight values represented 99.95% of the total sum.

**Figure 4:**
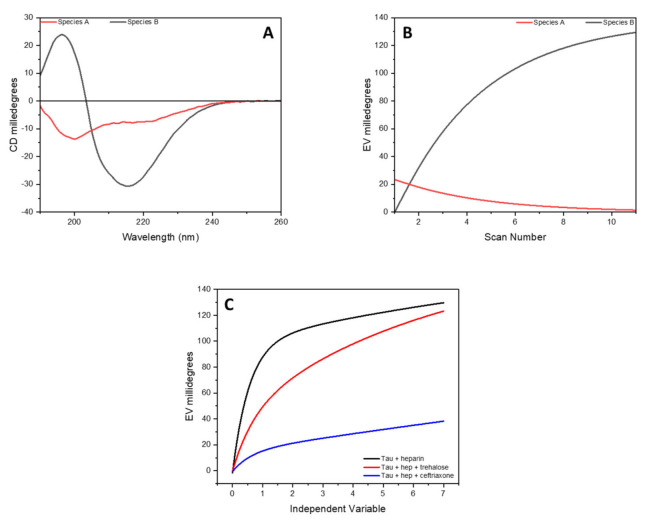
SVD analysis of complete data set of CD spectra of tau peptide for the conformational conversion of peptide induced by low-molecular-weight heparin. (**A**) CD spectra of the initial (specie A, in red) and final (specie B, in black) conformation of tau peptide in presence of 0.25 equivalents of heparin; (**B**) the time-dependent amplitude of the two species of tau peptide; (**C**) influence of trehalose or ceftriaxone on the conformational conversion of tau peptide induced by heparin.

To extract the spectra of these two species in the data set, a kinetic model based on the conversion of A into B (A→B) was used along with SVD. The determined spectra of A and B species as well as the kinetics of the structural transformation are reported in Figure 4 and correspond to the CD spectra of the peptide at time zero (specie A) and after 7 h of incubation (specie B).

To assess whether trehalose or ceftriaxone could interfere with or modulate the conformational conversion of tau peptide from unordered to β-sheet induced by heparin, CD experiments were carried out, monitoring the CD as a function of time upon the addition of either trehalose or ceftriaxone to the mixture of tau peptide with heparin.

The addition of six equivalents of trehalose to the peptide–heparin solution strongly modified the overall CD profile shape (Figure 2C) compared to that observed in presence of the heparin alone (Figure 2B). In particular, the CD spectrum recorded after 7 h of incubation at 25 °C showed two negative bands at 208 and 217 nm, while the positive band at 197 nm shifted to lower wavelengths (Figure 2C), resembling that of an α-helix structure. Additionally, in the presence of trehalose, any further change in the CD spectrum was observed after 7 h of incubation at 25 °C. The secondary structure estimation (SSE) analysis confirmed that in the presence of trehalose, the conformational conversion of tau peptide induced by heparin was strongly modified. Indeed, as reported in Table 1 and in Figure 3, at the end of the incubation time tau peptide adopted an α-helix secondary structure (48.6%), while the β-sheet content (20.4%), exclusively due to antiparallel structure, was similar to that estimated for the native peptide in the absence of heparin.

To evaluate the influence of trehalose on the conformation of tau peptide, six equivalents of trehalose were added to a peptide solution in the absence of heparin and incubated for 7 h at 25 °C. As shown in Figure S3 in the Supplementary Materials, no CD changes were observed as a function of time, confirming that trehalose did not affect the tau peptide conformation.

Even in the presence of heparin and trehalose, an isodichroic point was observed at about 205 nm, suggesting the presence of a single-phase transition from the initially unordered structure and the ordered conformation adopted by tau peptide after 7 h of incubation. The SVD analysis (Figure S4 in Supplementary Materials) of this set of CD spectra showed how even in the presence of heparin and trehalose, the contribution of each basis spectrum decreased rapidly, and only the first two spectra were used for the analysis (the sum of the squares of these values corresponds to 99, 98% of the entire sum), confirming the presence of an equilibrium between two species. It was therefore possible to use the previously described kinetic model (A→B) in order to determine the CD spectra of the species present at time 0 and at the end of the conformational transition of tau peptide, as well as the kinetics of the conversion (Figure S5 in Supplementary Materials).

Similarly, the effects of ceftriaxone on conformational conversion induced by heparin were evaluated. The set of CD spectra recorded in the presence of both heparin and ceftriaxone was reported in Figure 2D. The CD spectrum recorded at the end of the conformational transition closely resembled that acquired in the presence of heparin alone, with a strong positive band at 196 nm, a broad negative band centered at about 212 nm and an isodichroic point at 204 nm. Even in the presence of ceftriaxone, any significant change in the CD spectrum of tau peptide in the presence of heparin were observed after 7 h (data not shown), as well as any effect of ceftriaxone upon the addition to the peptide alone (Figure S6 in Supplementary Materials). The SSE at the end of the incubation time revealed a decrease in the content of ordered structures (α-helix and β-sheet) and an increment in the contribution of unordered structure compared to that of the sample incubated in the presence of heparin alone (Table 1 and Figure 3).

The SVD analysis (Figure S7 in Supplementary Materials) of this set of spectra indicated that in the presence of ceftriaxone, the system can also be ascribed as a single-phase transition with a kinetic model based on two species (Figure S8 in Supplementary Materials).

A comparison of the time-dependent amplitude of the appearance of specie B determined via SVD analysis is reported in Figure 4, where it can be seen how, in the presence of trehalose or ceftriaxone, a decrease in the conversion rate of the tau peptide was detected, suggesting a protective role of these two molecules toward the activity of heparin.

**Figure 5:**
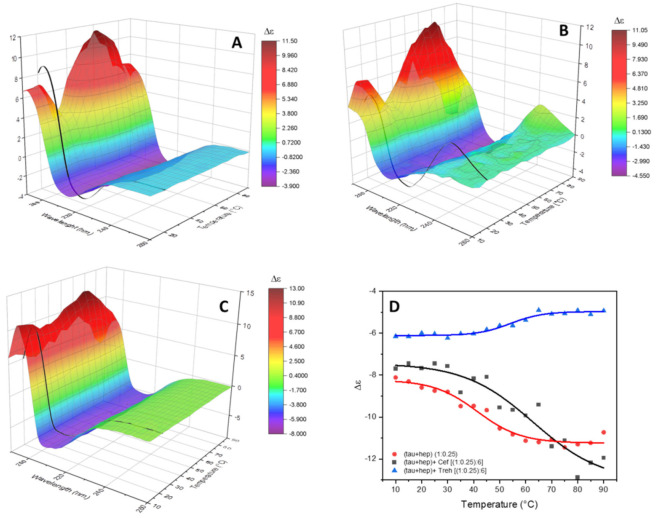
Temperature melting experiments. SRCD spectra in the 10–90 °C range recorded at 5 °C steps, represented as 3D surface map, of tau peptide in presence of low-molecular-weight heparin (**A**), of heparin and ceftriaxone (**B**) or of heparin and trehalose (**C**). In black, the SRCD spectrum of the analyzed solution recorded cooled back at 20 °C after the temperature ramping. (**D**) Melt curves built plotting the ellipticity value at 215 nm of tau peptide in presence of heparin alone (red line) or heparin and ceftriaxone (black line) or at 208 nm for heparin and trehalose (blue line).

The thermal stability of the product of the conformational conversion of tau peptide induced by heparin with and without trehalose was evaluated. The change in the CD spectra as a function of temperature were visually evident from both melt series spectra (Figure 5A–C) and melt curves obtained by plotting the measured molar circular dichroism values of the negative bands versus temperature (Figure 5D).

In the presence of heparin alone (Figure 5A), the set of CD spectra were characterized by an increased intensity of the negative band at 215 nm upon increased temperature, and the positive band at 197 nm showed a biphasic trend, initially decreasing up to 30 °C, then increasing up to 60 °C and then decreasing again. The secondary structure estimation analysis (Figure S9 in Supplementary Materials) shows the presence of more conformational transitions of tau peptide upon the increased temperature. The melt curve obtained, plotting the intensity of the negative band at 215 nm versus the temperature, enabled the determination of T*m* of 40 °C for the tau peptide with heparin.

In the presence of ceftriaxone, very similar melt series spectra were observed (Figure 5B), while the secondary structure estimation analysis showed a constant increase in the contribution of the unordered structure upon increasing the temperature (Figure S9B in Supplementary Materials). The T*m* of tau peptide with both heparin and ceftriaxone calculated using the intensity of the negative band at 215 nm increased to 62 °C. This increment in the T*m* value confirmed the presence of an interaction between tau peptide and ceftriaxone that stabilized the secondary structure of peptide.

The set of CD spectra recorded as a function of temperature in presence of both heparin and trehalose (Figure 5C) were characterized by a decrease in the intensity of the negative band at 208 nm upon increasing the temperature, while the estimation of secondary structure content (Figure S9C in Supplementary Materials) showed a slight decrease in the amount of α-helix conformation. An increased T*m* value of tau peptide with heparin and trehalose of 58 °C was indicative of binding interactions between trehalose and tau peptide, hence stabilizing the peptide conformation against aggregation.

### 2.2. Transmission Electron Microscopy

**Figure 6:**
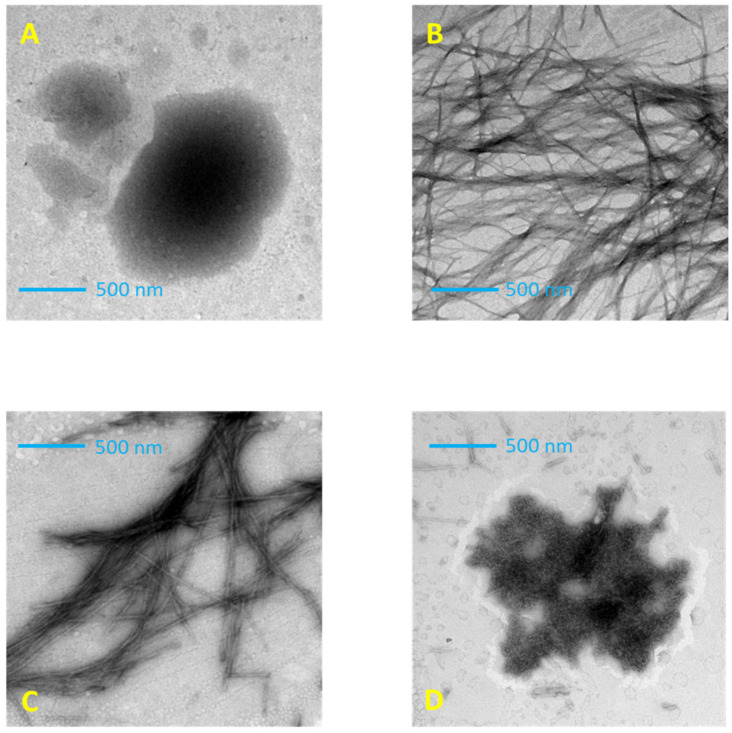
Transmission electron microscopy (TEM) imaging of tau peptide (70.9 µM) in 10 mM TRIS-HCl buffer, pH 7.4 after 7 h incubation at 25 °C. (**A**) Tau peptide alone; (**B**) in presence of 0.25 molar equivalents of low-molecular-weight heparin; (**C**) in presence of 0.25 molar equivalents of heparin and 6 molar equivalents of ceftriaxone; (**D**) in presence of 0.25 molar equivalents of heparin and 6 molar equivalents of trehalose.

The morphology of tau peptide was evaluated using negative staining transmission electron microscopy (TEM). As shown in Figure 6A, incubation with shaking of the tau peptide CD solution alone induces poor aggregation, and few amorphous aggregates were observed after one week of incubation (data not shown) according to the previous work of Mandelkow et al. [35].

The TEM analysis of peptide–heparin CD solution incubated with shaking at 25 °C revealed a mesh of interwoven filaments with a typical amyloid morphology consisting of networks of long, unbranched fibers greater than 500 nm (Figure 6B), confirming the ability of heparin to induce peptide aggregation and fibrillation, acting as a structured template for peptide self-assembly [36].

The TEM analysis of the peptide–heparin solution with added ceftriaxone still showed the presence of fibrillar structures, but with different morphologies compared to those previously described. Indeed, in the presence of ceftriaxone, the filament density was reduced, and the filaments were sparsely distributed. These remaining filaments were considerably shorter than the untreated fibrils and had greater thicknesses (Figure 6C). The TEM images concur with the CD indicating that ceftriaxone induces a different conformation of the tau peptide, resulting in modification of the fibril formation.

On the other hand, the presence of trehalose strongly modified the morphology of tau peptide aggregates. The addition of trehalose induced the disappearance of fibrillar structures with the appearance of amorphous aggregates (Figure 6D), confirming the CD results that indicate the absence of β-sheet structures. However, these aggregates differ in shape and size from those observed for the tau peptide alone (Figure 6A) and are consistent with the different secondary structure determined by CD.

## 3. Materials and Methods

### 3.1. Peptide Synthesis

Tau peptide was synthesized via solid phase peptide synthesis (SPPS) using Fmoc/DIC/Oxyma chemistry [37], which was carried out automatically on a Biotage^®^ Syro Wave™ Automated Microwave and Parallel Peptide Synthesizer (Biotage AB, Uppsala, Sweden) controlled by “SyroXP peptide” software. The N-terminal was acetylated via treatment with acetic anhydride, and successively the peptide was detached from the resin via treatment with TFA in the presence of triisopropylsylane and water as scavengers. This procedure achieved the simultaneous removal of sidechain-protecting groups. The crude peptide was precipitated via the addition of diethyl ether, and then the crude peptide was purified via RP-HPLC using a Dionex Vydac C18, 300 Å, 10 µ, 22 × 250 mm column (Thermo Fisher Scientific, Sunnyvale, CA, USA) mounted on a preparative Shimadzu HPLC system (Kyoto, Japan) equipped with LC-8A pumps, SLC-8A controller, an SPD-6A spectrophotometric detector and an ERC-3562 ERMA degasser (Erma, Tokyo, Japan).

The identity and purity of the synthesized peptide was determined via LC-ESI-MS analysis conducted using an Agilent 1260 Infinity II system equipped with a 6130 Quadrupole LC-MS analyzer.

### 3.2. Synchrotron Radiation Circular Dichroism

The secondary structure of tau peptide (70.9 µM) in 10 mM TRIS-HCl buffer, pH 7.4, alone or in the presence of low-molecular-weight heparin (0.25 equivalents), in the presence or absence of 6 equivalents of trehalose or ceftriaxone, were monitored for up to 7 h by acquiring far-UV SRCD spectra in the 190–260 nm range at Module B end station of Diamond Light Source Ltd. (Harwell Science and Innovation Campus, Didcot, UK) Beamline B23, using a 0.1 cm pathlength Suprasil quartz cuvette (Hellma Analytics, Müllheim, Germany), with 0.2 nm data pitch, 1 nm bandwidth, 1 s digital integration time and scan speed of 39 nm/min.

SRCD melting experiments on the different tau solutions in the absence or presence of 6 molar equivalents of alternatively trehalose or ceftriaxone after 7 h of incubation at 25 °C were performed in the 10–90 °C temperature range with 5 °C temperature increases and 3 min of equilibration time. After the measurement at 90 °C, the solutions were allowed to cool back to room temperature, and an additional SRCD spectrum was recorded after 8 min of equilibration time at 20 °C. Measurements were collected in the 190–260 nm range at Module B station of Diamond Light Source Beamline B23, using a 0.1 cm pathlength quartz cuvette (Hellma Analytics) with 0.2 nm data pitch, 1 nm bandwidth, 1 s digital integration time and scan speed of 39 nm/min.

Spectra were plotted using OriginPro2018 software (OriginLab Corporation, Northampton, MA, USA) and analyzed using the CDApps software [34] for Singular Value Decomposition analysis (SVD) and the web-available software BeStSel [32] to perform secondary structure estimation.

### 3.3. Transmission Electron Microscopy (TEM) Imaging

TEM images were taken on an FEI Tecnai G2 transmission electron microscope (Thermo Fisher Scientific, Waltham, MA, USA), operating at an excitation voltage of 100 kV. Specimen searching was carried out in diffraction mode at a low dose rate, which then quickly switched to image mode for immediate image acquisition. Sample preparation included staining with 1% uranyl acetate. Images were acquired using a Veleta digital camera (Olympus Soft Imaging System, Münster, Germany).

## 4. Conclusions

In this study, circular dichroism spectroscopy was utilized to monitor the conformational conversion of a short peptide, corresponding to the sequence 273–284 of tau protein and containing the PHF6* region involved in the protein aggregation, induced by low-molecular-weight heparin. Furthermore, the influence of small molecules, ceftriaxone and trehalose, of which we had previously demonstrated the antiaggregating properties toward α-synuclein and GFAP proteins, on the conformational conversion process was evaluated.

We reported experimental evidence that both these molecules, ceftriaxone and trehalose, interacted with tau peptide in the presence of heparin, increasing the T*m*, which is an indication of ligand binding interaction and the increased stability of the peptide toward aggregation, and more importantly, interfering with the peptide aggregation induced by heparin. As confirmed by TEM analysis, while the addition of ceftriaxone slightly modified the morphology of the peptidic aggregates, reducing the number of fibrils and increasing their diameter, the presence of trehalose removed the formation of fibrils by promoting α-helix conformation in tau peptide. Indeed, the TEM analysis revealed the presence of amorphous aggregates, morphologically different from those obtained with the native peptide in the absence of any additive (heparin and/or small molecules).

Overall, the data presented here confirm our previous results on the protective effects of trehalose toward protein aggregation and fibrillation, generalizing its effect on a wide range of proteins. It is important to note that trehalose is present in many fruits, vegetables, algae and processed foods. This work supports the need to investigate whether a diet richer in trehalose both from the intake of these foods or for the consumption of foods enriched with it might have an effect that protects or slows down the onset of pathologies linked to protein misfolding.

## Figures and Tables

**Table 1:** Percentage of secondary structure content of tau peptide after 7 h of incubation at 25 °C in presence of different molecules, along with the normalized root mean square error deviation (NRMSD) associated with the calculation.

	% Secondary Structure Estimation						
	**α**	**β_anti_**	**β_parallel_**	**β_tot_**	**Turn**	**Unordered**	**NRMSD**
**tau peptide**	15.1	22.5	0	22.5	14.3	48.1	0.024
**+ heparin**	36.5	7.2	34.6	41.8	0	21.7	0.048
**+ hep and treh**	48.6	20.4	0	20.4	5	26	0.017
**+ hep and cef**	14.2	15.5	10.8	26.3	12.4	47.1	0.037

## Supplementary Materials

The following supporting information can be downloaded at: https://www.mdpi.com/article/10.3390/ijms23062932/s1.
