# Supplementary material for: Effect of Trehalose and Ceftriaxone on the Stability of Aggregating-Prone Tau Peptide Containing PHF6* Sequence: An SRCD Study
Source: Int J Mol Sci. 2022 Mar 8;23(6):2932. doi: 10.3390/ijms23062932 (PMC8951053; doi:10.3390/ijms23062932)
Supplement: Supplementary file 1 [file ijms-23-02932-s001.zip › ijms-1596512-supplementary.pdf]

Effect of Trehalose and Ceftriaxone on the Stability of Aggregating-Prone Tau Peptide Containing PHF6* Sequence: An SRCD Study

Supplementary Information

Claudia Honisch[1,2], Federica Torni[1], Rohanah Hussain[3], Paolo Ruzza[1]* and Giuliano Siligardi[3]*

[1] Institute of Biomolecular Chemistry of CNR, Padua Unit, via Marzolo,1, 35131, Padova, Italy.

E-mail: paolo.ruzza@cnr.it; c.honisch@icb.cnr.it; tornifederica@gmail.com

[2] Department of Chemical Sciences, University of Padua, via Marzolo, 1, 35131, Padova, Italy.

E-mail: claudiahonisch@phd.unipd.it

[3] Diamond Light Source Ltd, Harwell Science and Innovation Campus, Didcot, Oxfordshire, OX11 0DE, United Kingdom.

E-mail: giuliano.siligardi@diamond.ac.uk.; rohanah.hussain@diamond.ac.uk

*Correspondence: giuliano.siligardi@diamond.ac.uk and paolo.ruzza@cnr.it.

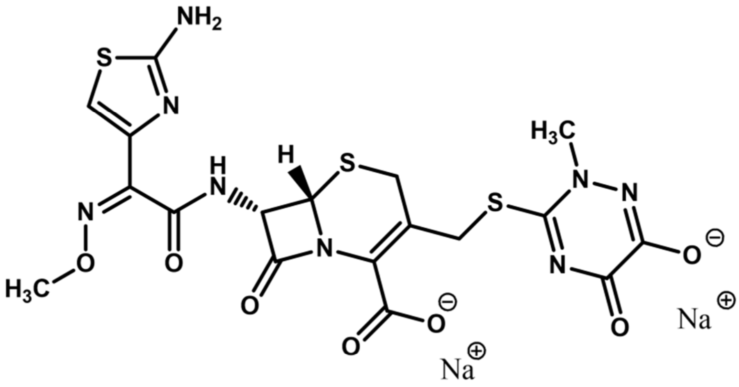

Ceftriaxone disodium salt

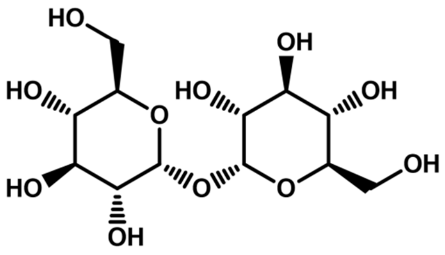

Trehalose

Figure S1. Chemical structure of the investigated small molecules ceftriaxone and trehalose.

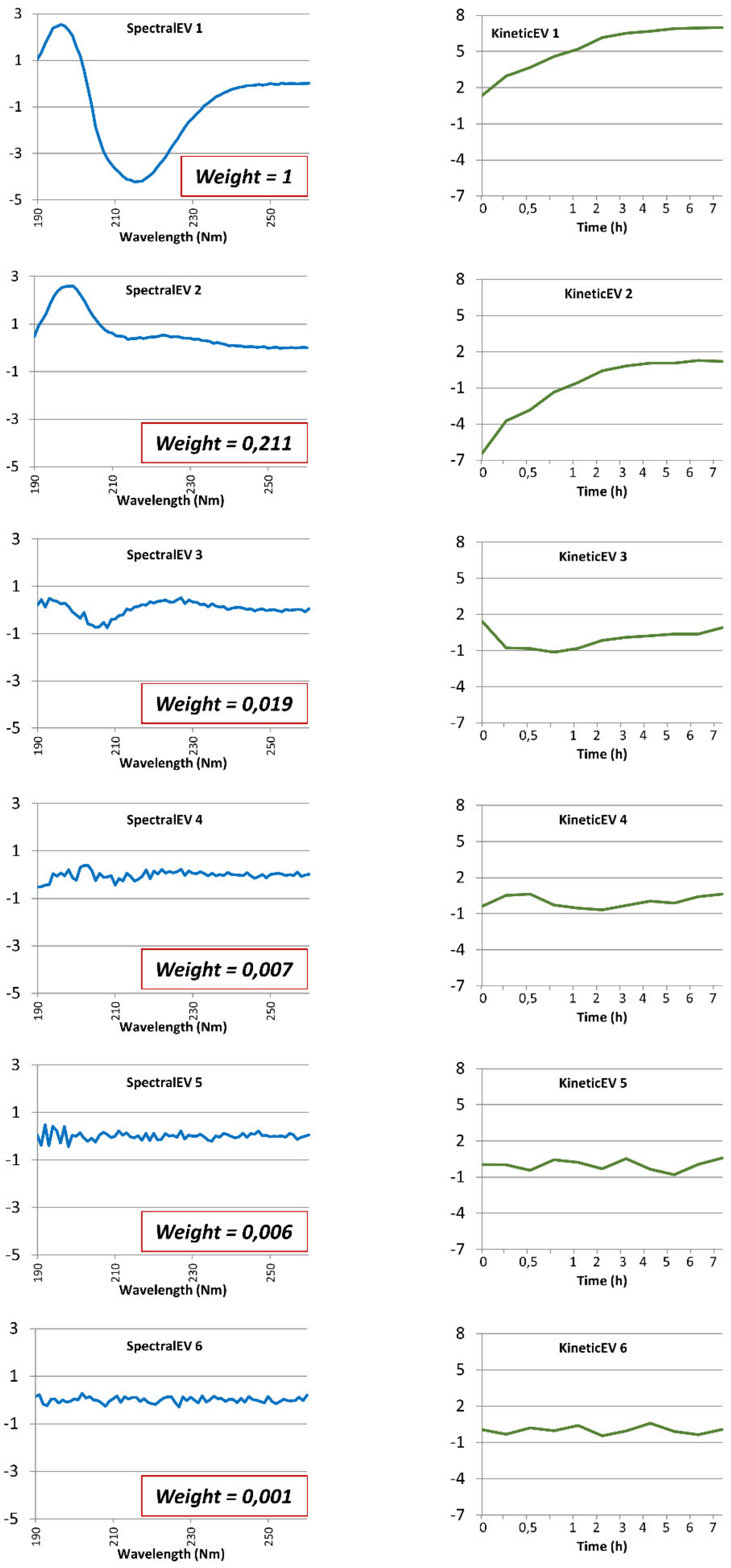

Figure S2. Singular value decomposition analysis of the complete data set of CD spectra of tau peptide incubated in presence of 0.25 equivalents of heparin up to 7 hours. Left column shows the spectral features and right column the time-dependent amplitude of corresponding feature. The ranking of the elements are reported in the insert box.

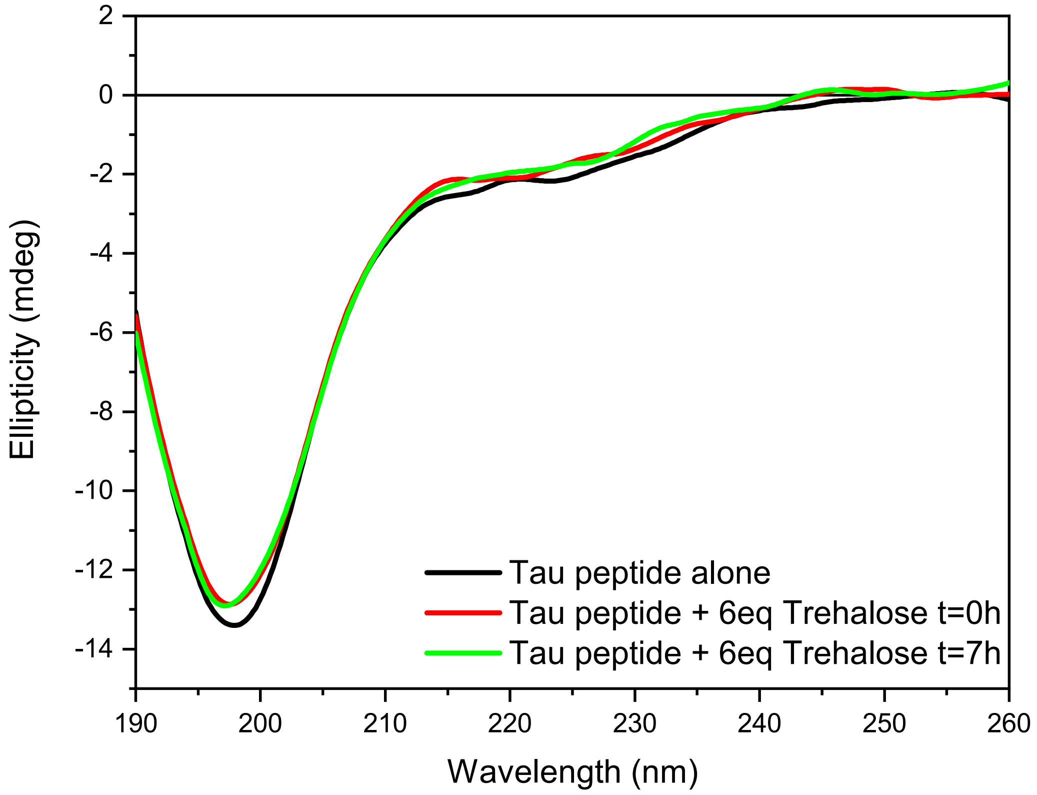

Figure S3. Far-UV SRCD spectra of tau peptide 70.9 µM alone (black) or in presence of 6 molar equivalents of trehalose (red at time zero, green after 7 hours incubation), in 10 mM TRIS-HCl buffer, pH 7.4. Spectra were acquired in the 190-260 nm region at Module B endstation of Diamond Light Source Beamline B23, using a 0.1 cm Suprasil quatz cuvette. Scan speed was 39 nm/min and bandwidth was 1 nm.

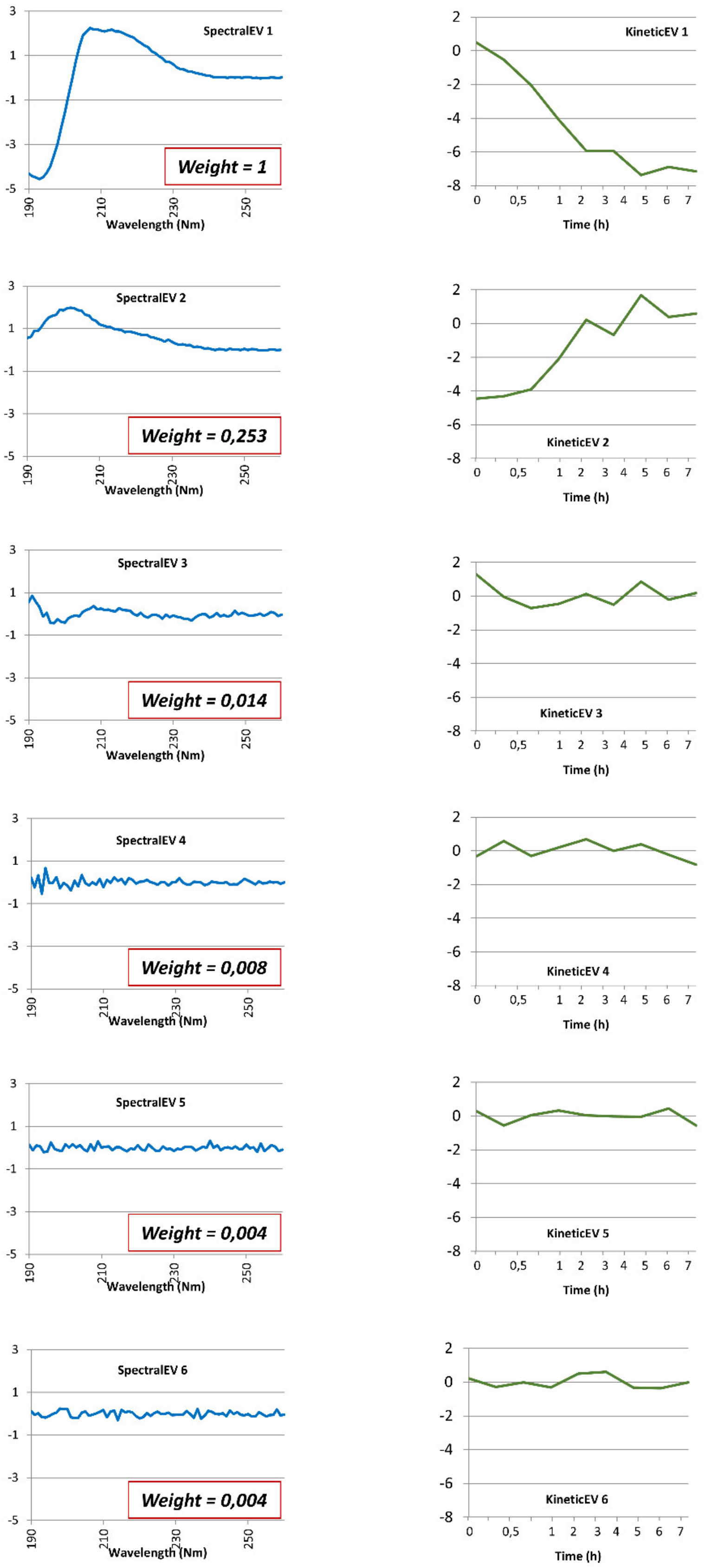

Figure S4. SVD decomposition analysis of the CD spectra set acquired for tau peptide incubated in presence of 0.25 molar equivalents of heparin and 6 molar equivalents of trehalose up to 7 hours. Left column: basis spectra representing the independent spectral features; right column: kinetic traces for each of these features; in the insert box the ranking value. Analysis performed using CDApps software (Hussain, R. et al. *J Synchrotron Radiat.* **2015,** 22, 862).

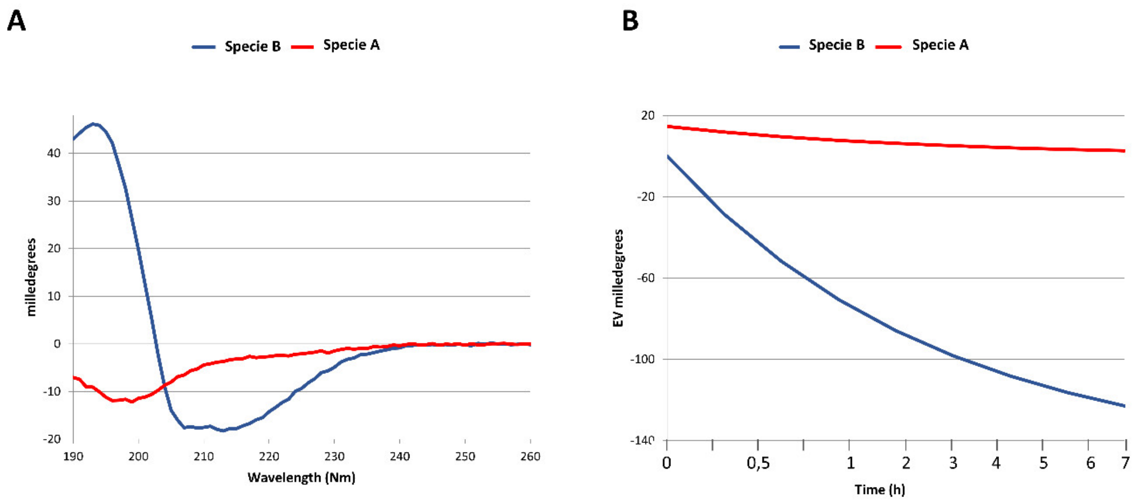

Figure S5. SVD analysis of complete data set of CD spectra of tau peptide for the conformational conversion of peptide incubated in presence of 0.25 equivalents of heparin and 6 equivalents of trehalose up to 7 hours. (A) CD spectra of the initial (specie A, in red) and final (specie B, in blue) conformation. (B) The time-dependent amplitude of the two species. Analysis performed using CDApps software (Hussain, R. et al. *J Synchrotron Radiat* **2015,** 22, 862).

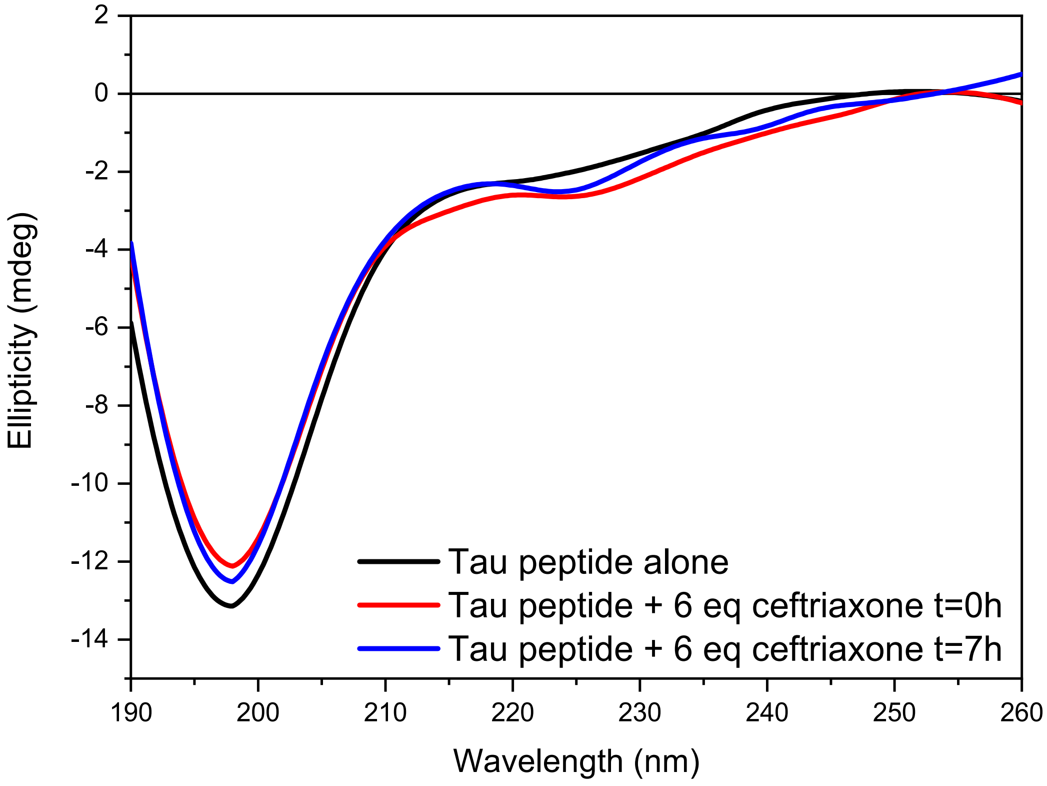

Figure S6. Far-UV SRCD spectra of tau peptide 70.9 µM alone (black) or in presence of 6 molar equivalents of ceftriaxone (red: at time zero, blue: after 7 hours incubation), in 10 mM TRIS-HCl buffer, pH 7.4. Spectra were acquired in the 190-260 nm region at Module B endstation of Diamond Light Source Beamline B23, using a 0.1 cm Suprasil quatz cuvette. Scan speed was 39 nm/min and bandwidth was 1 nm.

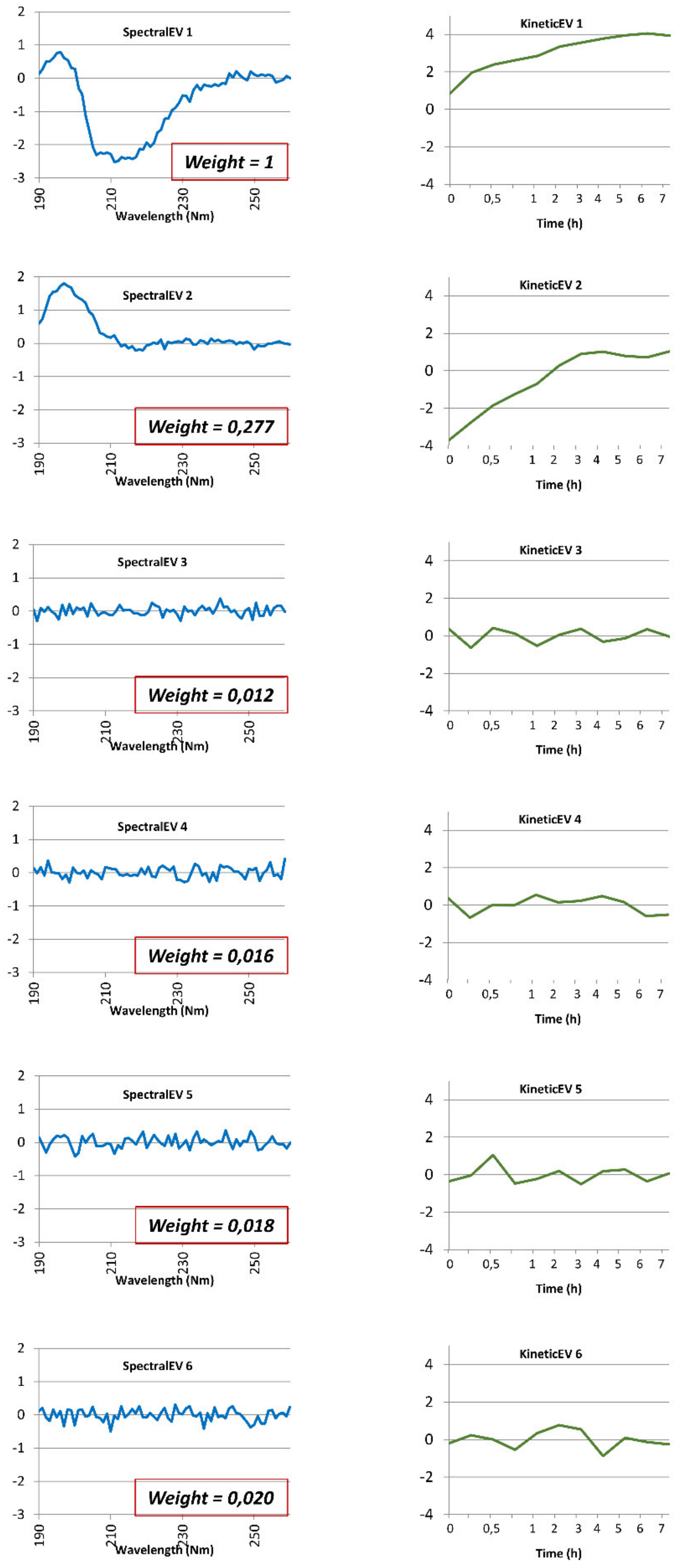

Figure S7. SVD decomposition analysis of the spectra acquired for tau peptide incubated in presence of 0.25 molar equivalents of heparin and 6 molar equivalents of ceftriaxone up to 7 hours. Left column: basis spectra representing the independent spectral features; right column: kinetic traces for each of these features; insert box: weight value. Analysis performed using CDApps software (Hussain, R. et al. *J Synchrotron Radiat* **2015,** 22, 862).

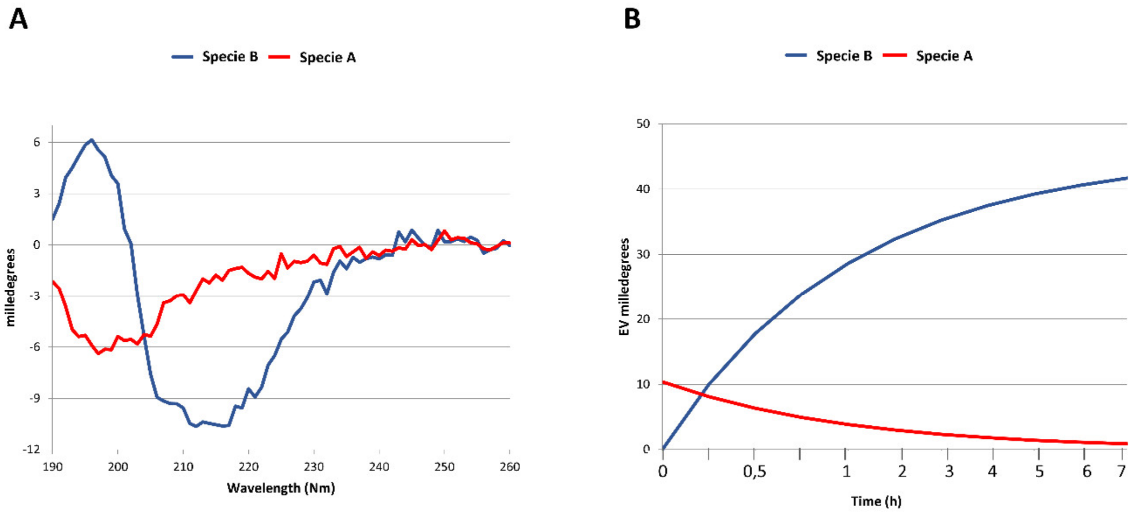

Figure S8. SVD analysis of complete data set of CD spectra of tau peptide for the conformational conversion of peptide incubated in presence of 0.25 equivalents of heparin and 6 equivalents of ceftriaxone up to 7 hours. (A) CD spectra of the initial (specie A, in red) and final (specie B, in blue) conformation. (B) The time-dependent amplitude of the two species. Analysis performed using CDApps software (Hussain, R. et al. *J Synchrotron Radiat* **2015,** 22, 862).

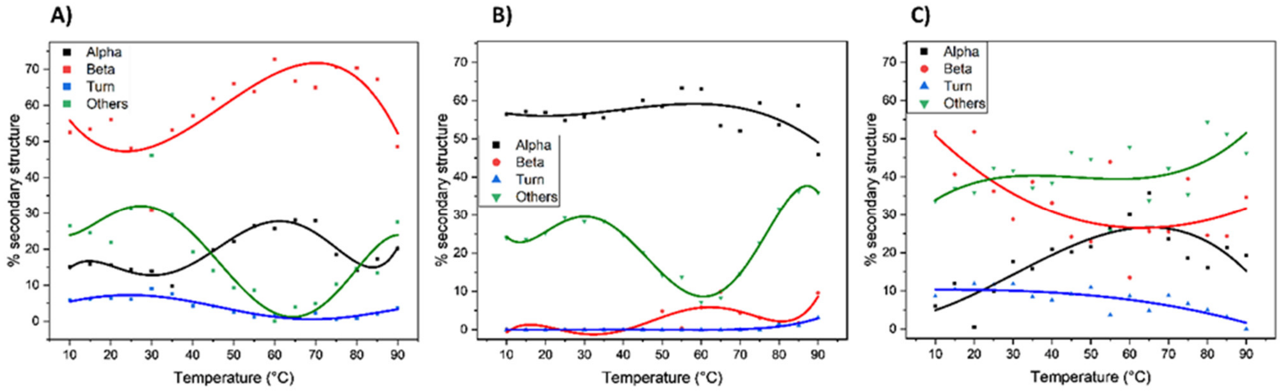

Figure S9. Plot of secondary structure composition trend during the melting experiment (α-helix in black, β-strand in red, turns in blue and unordered in green). CD spectra for A) tau peptide (70.9 µM) in 10 mM TRIS-HCl buffer, pH 7.4, added of 0.25 equivalents of low-molecular-weight heparin, for B) tau peptide in presence of heparin and 6 equivalents of trehalose, and for C) tau peptide (70.9 µM) in presence of heparin and 6 equivalents of ceftriaxone, were acquired in the 10-90°C range, with 5°C step, allowing 3 minutes equilibration time before each acquisition, at Module B end station of Diamond Light Source Beamline B23, using a 0.1 cm Suprasil quatz cuvette. Scan speed was 39 nm/min and bandwidth was 1 nm. Secondary structure estimation was performed using the BeStSel web application [Micsonai et al, *Nucleic Acids Res*, 46:W315-22, **2018**. Micsonai et al, *PNAS*, 11:E3095-130, **2015**].

A)

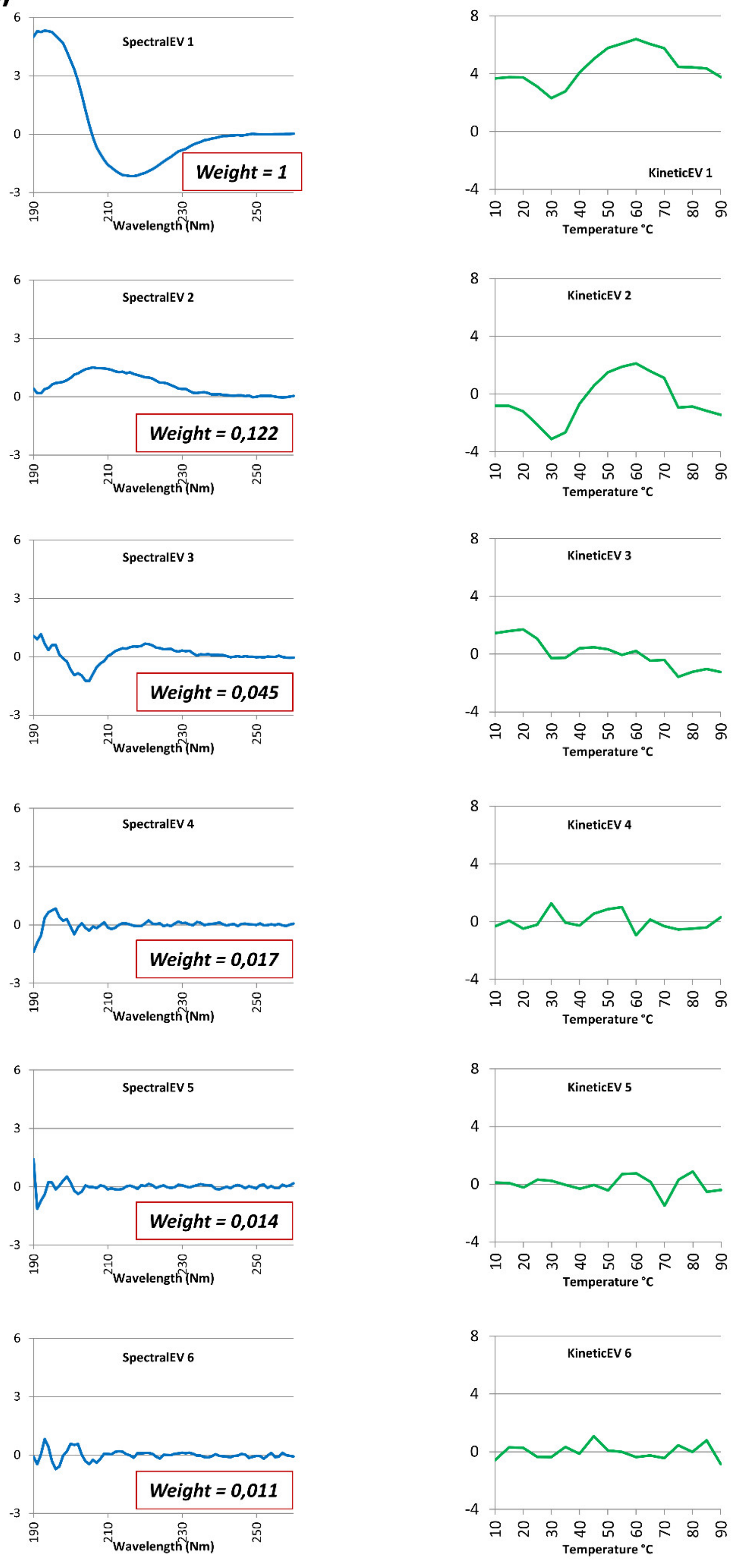
SpectralEV 1
Weight = 1
Wavelength (Nm)
KineticEV 1
Temperature °C
SpectralEV 2
Weight = 0,122
Wavelength (Nm)
KineticEV 2
Temperature °C
SpectralEV 3
Weight = 0,045
Wavelength (Nm)
KineticEV 3
Temperature °C
SpectralEV 4
Weight = 0,017
Wavelength (Nm)
KineticEV 4
Temperature °C
SpectralEV 5
Weight = 0,014
Wavelength (Nm)
KineticEV 5
Temperature °C
SpectralEV 6
Weight = 0,011
Wavelength (Nm)
KineticEV 6
Temperature °C

B)

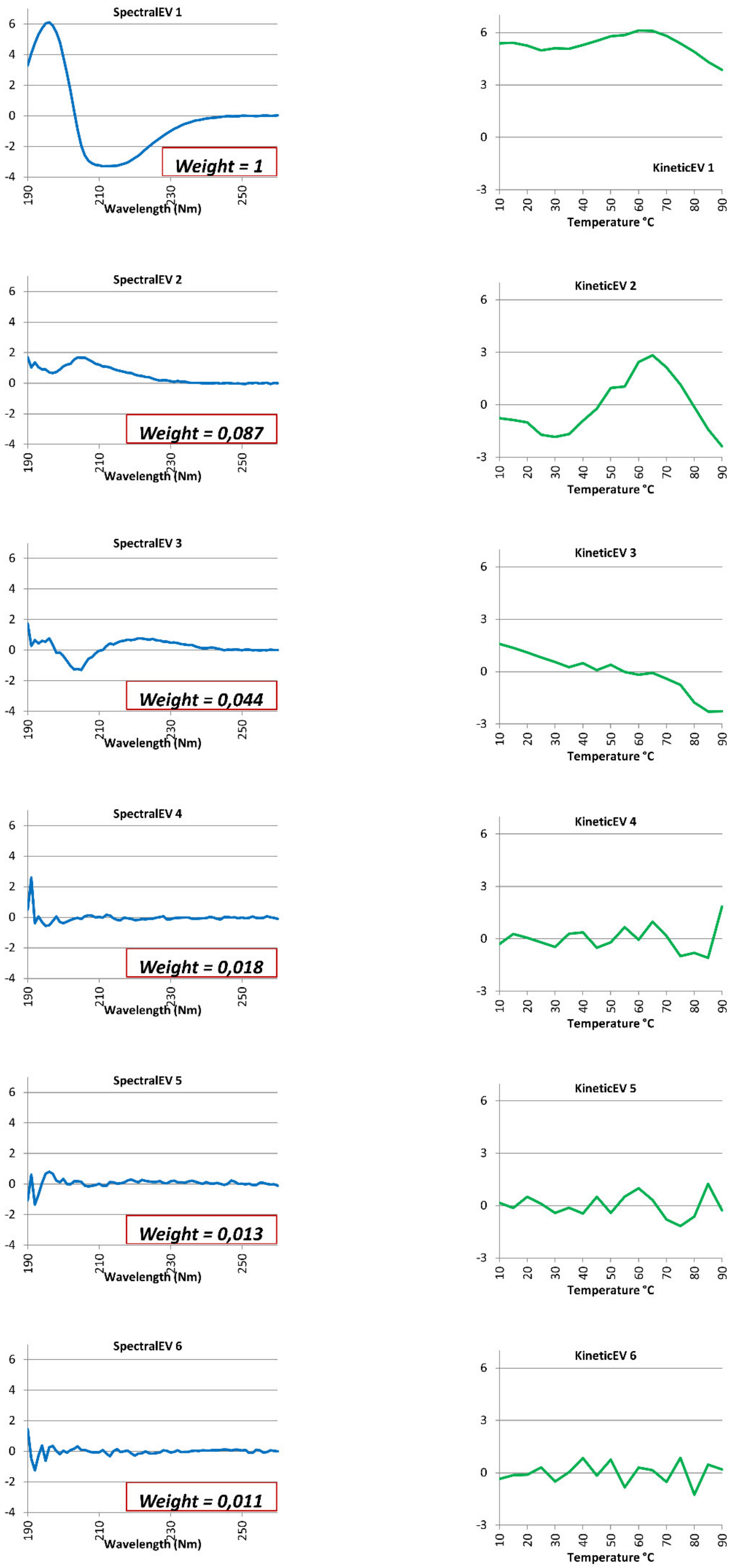
SpectralEV 1
Weight = 1
Wavelength (Nm)
KineticEV 1
Temperature °C
SpectralEV 2
Weight = 0,087
Wavelength (Nm)
KineticEV 2
Temperature °C
SpectralEV 3
Weight = 0,044
Wavelength (Nm)
KineticEV 3
Temperature °C
SpectralEV 4
Weight = 0,018
Wavelength (Nm)
KineticEV 4
Temperature °C
SpectralEV 5
Weight = 0,013
Wavelength (Nm)
KineticEV 5
Temperature °C
SpectralEV 6
Weight = 0,011
Wavelength (Nm)
KineticEV 6
Temperature °C

C)

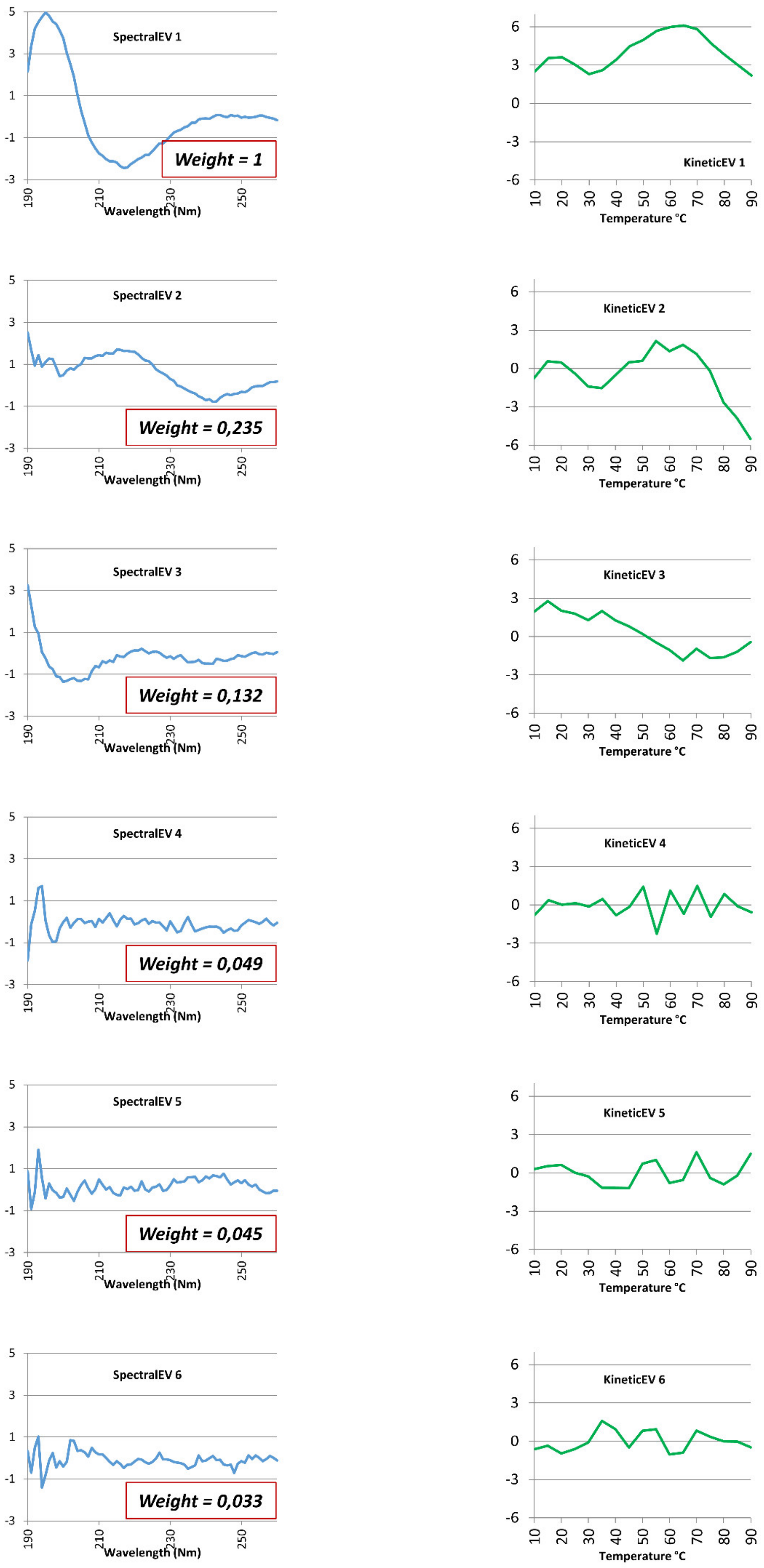

SpectralEV 1
Weight = 1
Wavelength (Nm)
KineticEV 1
Temperature °C
SpectralEV 2
Weight = 0,235
Wavelength (Nm)
KineticEV 2
Temperature °C
SpectralEV 3
Weight = 0,132
Wavelength (Nm)
KineticEV 3
Temperature °C
SpectralEV 4
Weight = 0,049
Wavelength (Nm)
KineticEV 4
Temperature °C
SpectralEV 5
Weight = 0,045
Wavelength (Nm)
KineticEV 5
Temperature °C
SpectralEV 6
Weight = 0,033
Wavelength (Nm)
KineticEV 6
Temperature °C

Figure S10. SVD decomposition analysis of the melting experiments(10-90°C range at 5°C steps). A) Tau peptide incubated in presence of 0.25 molar equivalents of heparin; B) Tau peptide in presence of heparin and 6 molar equivalents of trehalose, and C) Tau peptide in presence of heparin and 6 molar equivalents of ceftriaxone. For each, left column: basis spectra representing the independent spectral features; right column: kinetic traces for each of these features; insert box: weight value. Analysis performed using CDApps software (Hussain, R. et al. *J Synchrotron Radiat* **2015,** 22, (3), 862-862.)
